# Why Do Kestrels Soar?

**DOI:** 10.1371/journal.pone.0145402

**Published:** 2015-12-21

**Authors:** Jesús Hernández-Pliego, Carlos Rodríguez, Javier Bustamante

**Affiliations:** 1 Department of Wetland Ecology, Estación Biológica de Doñana (EBD-CSIC), c/Américo Vespucio s/n, 41092 Seville, Spain; 2 Department of Conservation Biology, Estación Biológica de Doñana (EBD-CSIC), c/Américo Vespucio s/n, 41092 Seville, Spain; INIBIOMA (Universidad Nacional del Comahue-CONICET), ARGENTINA

## Abstract

Individuals allocate considerable amounts of energy to movement, which ultimately affects their ability to survive and reproduce. Birds fly by flapping their wings, which is dependent on the chemical energy produced by muscle work, or use soaring-gliding flight, in which chemical energy is replaced with energy harvested from moving air masses, such as thermals. Flapping flight requires more energy than soaring-gliding flight, and this difference in the use of energy increases with body mass. However, soaring-gliding results in lower speeds than flapping, especially for small species. Birds therefore face a trade-off between energy and time costs when deciding which flight strategy to use. Raptors are a group of large birds that typically soar. As relatively light weight raptors, falcons can either soar on weak thermals or fly by flapping with low energy costs. In this paper, we study the flight behavior of the insectivorous lesser kestrel (*Falco naumanni*) during foraging trips and the influence of solar radiation, which we have adopted as a proxy for thermal formation, on kestrel flight variables. We tracked 35 individuals from two colonies using high frequency GPS-dataloggers over four consecutive breeding seasons. Contrary to expectations, kestrels relied heavily on thermal soaring when foraging, especially during periods of high solar radiation. This produced a circadian pattern in the kestrel flight strategy that led to a spatial segregation of foraging areas. Kestrels flapped towards foraging areas close to the colony when thermals were not available. However, as soon as thermals were formed, they soared on them towards foraging areas far from the colony, especially when they were surrounded by poor foraging habitats. This reduced the chick provisioning rate at the colony. Given that lesser kestrels have a preference for feeding on large insects, and considering the average distance they cover to capture them during foraging trips, to commute using flapping flight would result in a negative energy balance for the family group. Our results show that lesser kestrels prioritize saving energy when foraging, suggesting that kestrels are more energy than time-constrained during the breeding season.

## Introduction

Movement, as a crucial process that determines individual life history, affects survival and reproduction. Animals allocate energy to support physiological and behavioral traits, but especially to move within a landscape (e.g. [1,2]). Most avian species move by flying, either through flapping or soaring-gliding. The majority of birds fly by flapping their wings which requires their muscles to convert chemical energy into work [3], although they have also evolved morphological and behavioral adaptations to take advantage of the energy available in moving air masses and to fly by soaring-gliding with little muscle work [4,5]. Some sea birds depend on strong winds to soar when flying large distances over ocean waters using dynamic soaring [6]. By contrast, terrestrial birds can exploit upward winds deflected by cliffs and ridges to fly without flapping their wings using slope soaring, or they can exploit rising air columns, also known as thermals, using thermal soaring [7]. Thermals are created by the differential heating by solar radiation of the soil surface and the air in contact with it. Birds circle up on thermals, increase their flight altitude and then glide down to the next thermal in a similar way to man-made gliders [8]. Birds are therefore able to substitute muscle power with kinetic or potential energy extracted from the environment when soaring-gliding.

Flight theory predicts that the power needed for a soaring-gliding flight is about 1.5 times the basal metabolic rate, whereas flapping flight requires several times more energy [9,10]. This statement has been verified in empirical studies on diverse flying species [11,12]. Additionally, the power needed for flapping flight increases steeply with body mass [10,13], at the same time as the mass-specific basal metabolic rate decreases [14]. Therefore, the difference in energy expenditure between the two flight strategies increases with body mass [15]. As a consequence, the heavier a bird is, the more energy efficient it is to adopt soaring-gliding over the flapping flight strategy [16]. The question then arises of why not all birds use the soaring-gliding flight strategy if it is so advantageous.

There are other morphological traits apart from body mass, such as wing shape or wingspan that affect bird flight performance and consequently can be critical in deciding which flight strategy to adopt [9,17]. But, in the case of thermal soaring, the answer may also lie in the spatial and temporal constraints imposed by this flight strategy that potentially offsets its energy advantage. Thermals are the result of convective processes between the earth’s surface and the air in contact with it, and do not develop uniformly over an heterogeneous landscape [18]. The spatial scale of thermal formation is of the order of hundreds or thousands of meters, which usually exceeds the home range of smaller bird species and consequently prevents them from using thermal soaring when searching for resources [19]. In addition, as thermals are weak over the sea, birds are forced to make detours over land when using soaring-gliding flights during migration, which in turn extends traveling time [20]. Furthermore, thermals are not permanently available because their formation depends on adequate weather conditions, which limits the time available to fly [21]. Thermal formation follows a daily pattern: it begins shortly after sunrise, increases in depth and intensity throughout the morning, peaks around noon, and then decreases towards sunset [18]. Soaring birds usually adapt their daily movements to this predictable pattern in order to exploit the thermals available in an efficient way and thus fly with reduced costs [22]. In addition, given the spatial and temporal pattern in thermal formation that soaring birds have to cope with, they fly at lower cross-country speeds when using thermal soaring than when flapping [3,15,23]. Birds therefore face a trade-off between energy and time costs when deciding which strategy to adopt when flying. As a general trend, large terrestrial birds use thermal soaring in order to reduce flight costs, whereas small birds use flapping flights as the energy benefits linked to soaring on thermals does not compensate for the time costs experienced [15].

Raptors are a representative group of large soaring birds. Within this group, falcons are relatively light with a low body mass and low wing loading [24]. These morphological characteristics allow them to soar on a wide range of thermal intensities [25] but also to fly by flapping with relatively low energy costs [16]. Falcons do not seem as constrained by thermal formation as larger raptors do, and they can fly throughout the entire day and even at night when thermals do not form [26–28]. Moreover, falcons are able to cross large bodies of water where thermals are weak or absent [29,30]. Accordingly, falcons have traditionally been considered flapping raptors with a preference for powered flight, without any need for thermals to fly [17,26,31–35]. Nevertheless, preliminary data on the foraging movement of the lesser kestrel, *Falco naumanni* (one of the smallest falcons in the world), showed higher than expected frequencies of soaring when individuals were commuting between the breeding colony and the foraging areas. We therefore designed a study to evaluate to what extent this species relies on thermal soaring for foraging.

Lesser kestrels are small insectivorous colonial falcons that breed in buildings and cliffs in steppe-like habitats, pastures and non-irrigated crops [36]. Throughout the breeding season, lesser kestrels continuously prospect the surroundings of their colonies to locate the ephemeral, concentrated, and unpredictable abundances of insects across a heterogeneous environment [37,38]. Because kestrels do not store prey items they have to return to the colony to provision their mates or chicks once they capture a prey. The soaring-gliding flight strategy would allow kestrels to reduce flight costs when searching for food during an energy-intensive period such as the breeding season [39]. In this study, we tracked individual lesser kestrels using high frequency GPS-dataloggers to investigate flight behavior along foraging trips during the breeding season. 1) We hypothesized that lesser kestrels would adopt the soaring-gliding flight strategy along foraging trips in suitable thermal conditions. 2) We expected individuals to increase flight altitude as thermals increase in depth and intensity throughout the day in order to obtain higher potential energy values during foraging trips. 3) We also expected individuals to use this potential energy gain to fly larger distances with reduced costs and to reach foraging patches located far from the colony, especially if prey availability is low close to the colony. Additionally, 4) we calculated power requirements for lesser kestrels to complete a foraging trip and the daily energy expenditure when adopting a pure flapping or a pure soaring-gliding flight strategy in order to evaluate the trade-off between the two flight strategies.

## Material and Methods

### Ethics Statements

The environmental authority (Dirección General de Gestión del Medio Natural y Espacios Protegidos, Junta de Andalucía) provided permits to access the study colonies and to attach GPS-dataloggers to this endangered species. The Doñana Biological Station Ethics Committee on Animal Experimentation (CEEA-EBD), the Bioethics Subcommittee of the Spanish National Research Council (CSIC) and the Consejería de Agricultura, Pesca y Desarrollo Rural (Junta de Andalucía) all reviewed the marking protocol and approved the research plan of the HORUS project.

### Study area

**Fig 1:**
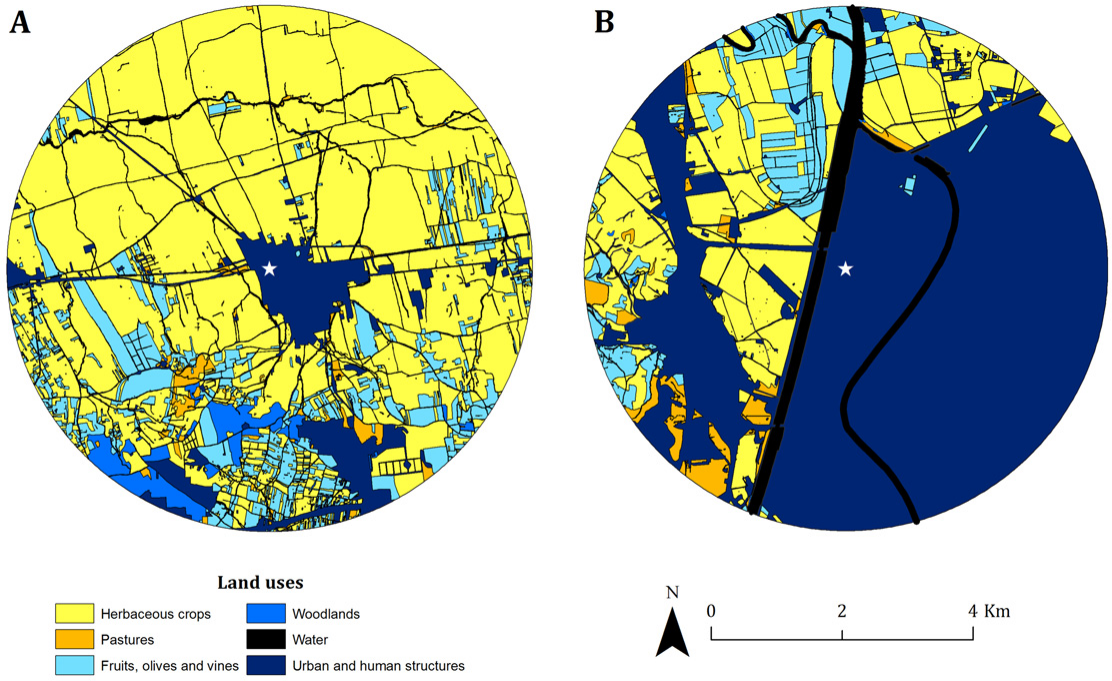
Land uses within a 4 km-buffer around the two study colonies: Silo colony (A) and EBD colony (B). The white star indicates the location of the colony in each panel. Good foraging habitats for the lesser kestrel are represented by shades of yellow and poor foraging habitats represented by shades of blue.

We studied lesser kestrels at two breeding colonies in the Guadalquivir river basin (southwestern Spain). The terrain is predominantly flat (20–240 m) but features some hills and escarpments and is dominated by arable crops [40], predominantly wheat and sunflower, although olive groves, fruit trees and vineyards are also present. The Silo colony is situated at a building with a grain elevator located in agricultural land, while the EBD colony is situated 50 km away on the roof of our research institute in Seville and dominated by urban land uses (Fig 1). At both sites, the lesser kestrels nest inside nest-boxes installed at both buildings.

### Field procedures

Lesser kestrel breeding pairs were monitored during the 2011–2014 breeding seasons. We attached GPS-dataloggers (GiPSy models 2, 4, and 5; Technosmart, Rome, Italy) with small batteries (90–100 mA) to the birds nesting in nest-boxes. GPS devices were fixed to the birds’ backs using a micro back-pack harness supplied by Marshall Radio Telemetry (North Salt Lake, Utah, U.S.A.) or a similar hand-made harness formed by a carbon fiber plate and a 4 mm wide Teflon ribbon (Bally Ribbon Mills, Pennsylvania, U.S.A.). GPS-dataloggers were covered with a protective thermoretractable case. The total mass of the equipment (harness + GPS + battery) was about 6 g and never exceeded the 5% of the lesser kestrel’s mean body mass, which is within the recommended limits for flying animals [41]. To get the birds used to the harness and the GPS device, we fixed a dummy GPS-datalogger with the same weight to the harness at least a week before fixing the real device and starting to record the birds’ movement (see details of the procedure in Hernández-Pliego *et al*. [42]).

We attached GPS-dataloggers to 39 individual lesser kestrels during the study period, but were unable to recover tracking data from 4 of them. Finally, we obtained a total of 825,365 GPS-fixes from 35 individuals (17 females and 18 males). Some of them were tracked during two (8 individuals) or three (1 individual) breeding seasons. We configured the GPS-dataloggers at five different sampling frequencies: one fix per second, one fix per minute, or every 3, 5, and 10-minutes. Since the GPS stored the data in the logger, we had to recapture the individuals to recover the data. A new full-powered device was then deployed before releasing the individual to be able to continue tracking. The kestrels were captured when they entered the nest-boxes using remote-controlled sliding doors. Individuals were telemetered during a mean of 55.86 ± 30.72 days per breeding season, range 6–100 days; they were recaptured a mean of 5.16 ± 2.44 times per year, range 2–11 (n = 45). Data were collected during daylight hours (5 to 20 h UTC) during the breeding season (March–July). We removed the harnesses from the kestrels at the end of each breeding season. The tracking data can be consulted on Movebank (www.movebank.org) (DOI: 10.5441/001/1.sj8t3r11).

### Weather data

Because solar radiation is the engine for thermal updraft, it can be considered a proxy for thermal formation [8,43]. We obtained solar radiation data from the agroclimatic station network of the Andalusian Agricultural Department (http://www.juntadeandalucia.es/medioambiente/servtc5/WebClima/), collected at the meteorological station of La Palma del Condado by a Skye SP1110 pyranometer every 30 minutes. The station is situated 192 m above sea level, 3 km from the Silo colony and 48 km from the EBD colony.

It is possible to estimate directly thermal and orographic uplift from meteorological models, but the models available provide these data at low temporal (6 hours) and spatial (0.75 degrees) resolutions. We obtained estimates of thermal and orographic uplift throughout the study period from the Movebank Environmental Data Automated Track Annotation (Env-DATA) system [44]. Thermal and orographic uplift estimates were calculated by reanalyzing weather data from the European Centre for Medium-Range Weather Forecasts (ECMWF). We obtained two orographic uplift estimates that were calculated using data from different digital elevation models (DEM): the Shuttle Radar Topography Mission (SRTM) 90-m DEM, and the Advanced Spaceborne Thermal Emission and Reflection Radiometer (ASTER) 30-m DEM. We did not expect slope soaring to be a frequent flight strategy for lesser kestrels in our study area which is mostly flat. Nonetheless, because of the existence of hills and escarpments that can deflect wind that can be exploited by individuals, we decided to evaluate the effect of orographic uplift on lesser kestrel flight behavior. Because of the temporal and spatial resolutions of the estimates, we had only 4 values per day of thermal and orographic uplift at the location of each colony.

### Flight variables

GPS locations were explored graphically using GIS (ArcGIS 10, ESRI, Redlands, California, U.S.A.) to identify individual foraging trips. We use the term foraging trip to refer to a set of consecutive locations of an individual kestrel which, start from the breeding colony and extend beyond 300 m and in which we are able to identify a foraging event (mostly clumped locations at low altitude above the ground with highly variable instantaneous speed). The details of the foraging trip identification and segmentation procedure can be found in Hernández-Pliego *et al*. [42]. The movements from the colony to the area where the foraging event took place and the return movement to the colony are referred to as commuting flights (outward and inward flight, respectively). Incomplete foraging trips, i.e. trips in which departure from or arrival at the colony was not recorded by the GPS were removed from statistical analyses. We also removed those foraging trips that started or finished at roosting sites away from the colony.

GPS devices provided the flight altitude and instantaneous speed for each location. We calculated the flight altitude above ground as the difference between the flight altitude recorded by the GPS and the topographic elevation obtained from a 10 m-resolution DEM obtained from the Andalusian Environmental Department (REDIAM, Junta de Andalucía, 2010–2011). We removed any position with low accuracy (less than 4 satellites, dilution of precision over 3, or positions with negative altitude values).

**Fig 2:**
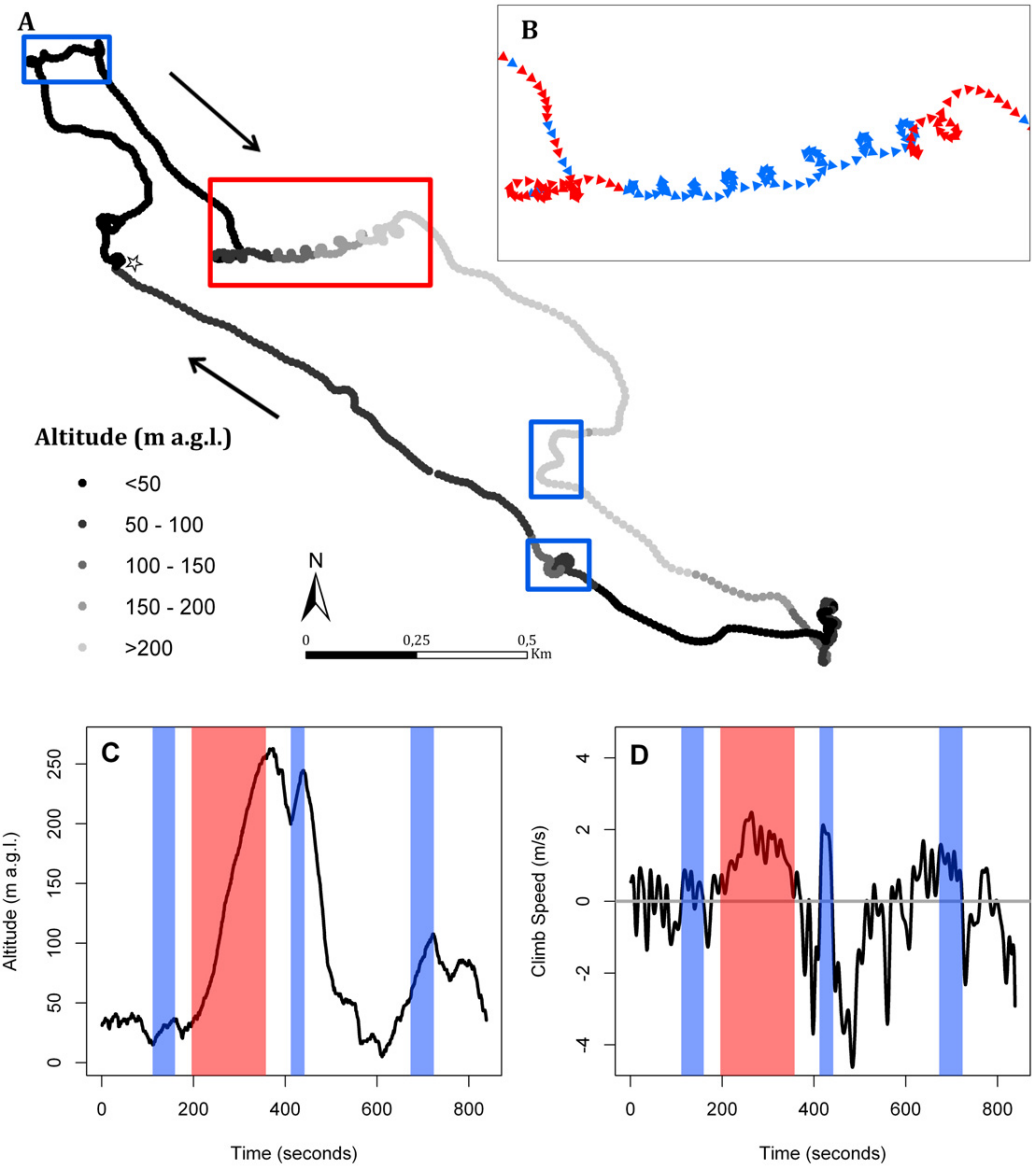
Track of a lesser kestrel foraging trip using thermal soaring along the commuting flights. (A) The white star represents the breeding colony. Each location of the path is colored according to flight altitude above ground level. Black arrows indicate movement direction. Red and blue boxes mark thermal soaring events. (B) Zoomed view of the thermal soaring event included in the red box. Locations are represented by triangles pointing to the direction of movement and its color indicates the circling direction either clockwise (red) or counterclockwise (blue). (C) Altitude and (D) climb speed profiles of the foraging trip. Red and blue shaded areas represent the thermal soaring events included in the boxes of panel A.

We analyzed commuting flights recorded at 1-second intervals in order to investigate the use of thermals by lesser kestrels. Thermal soaring events in these commuting flights recorded at 1-s are easily detectable through a circular flight path with an increase in flight altitude and positive climb speed (Fig 2) [8,16]. Soaring events were considered to be any flight segment with a circular pattern lasting more than 5 s, with positive vertical speed and that resulted in an increase in altitude of at least 10 m. To err on the conservative side, we only considered the climbing phase of the thermal soaring events for our analyses since the gliding phase might include flapping flights. For commuting flights in which we identified thermal soaring events, we calculated the following parameters indicative of the intensity or efficiency in the use of thermals by kestrels: (1) number of thermal soaring events and (2) accumulated ascent per horizontal distance covered; and (3) total ascent and (4) mean climb speed per thermal soaring event. Furthermore, we analyzed all foraging trips regardless of the sampling frequency at which they were recorded, from 1-second to 10-minutes, in order to study changes in the daily pattern of lesser kestrel flights. For these foraging trips, we calculated the following flight variables: (1) mean cross-country speed and (2) maximum flight altitude recorded per commuting flight; and (3) maximum distance from the colony and (4) duration of the foraging trip. To reduce the influence of outliers we used the third quartile of flight altitude as the maximum flight altitude because a single maximum value might be highly influenced by GPS altitude errors. In order to study the influence of solar radiation in all those flight variables, we used the value of solar radiation measured at the time rounded to the nearest half-hour when each commuting flight started. In foraging trips in which the outward flight was not recorded because the sampling frequency was longer than its duration, we calculated the time rounded to the nearest half hour when the first location was obtained and the solar radiation measured at that time. Additionally, to estimate the importance of thermal or topographic uplift in determining lesser kestrel foraging flight strategies, we built models to evaluate its influence on flight variables. As thermal and orographic uplift are estimated at a rough temporal scale (6-h intervals), we calculated the mean values of every flight variable obtained from commuting flights or foraging trips (depending on the variable) included in each of these intervals, and separately for each colony. We pooled all individuals and flights tracked at each colony during each 6-h interval to estimate the mean values.

### Energy expenditure

To calculate lesser kestrel flight power requirements we used Pennycuick’s Flight software version 1.24 (http://www.bristol.ac.uk/biology/people/colin-j-pennycuick). We considered a body mass of 130 g (the mean value for the species [45]) to carry out the calculations. We also assumed a mean wingspan of 0.68 ± 0.02 m (± standard deviation) and a mean wing area of 0.062 ± 0.0002 m^2^, based on data obtained from field measurements (authors’ unpub. data, n = 5) in line with Pennycuick’s procedure [3]. We estimated the energy needed for a lesser kestrel to perform a foraging trip when adopting a pure flapping or a pure soaring-gliding flight strategy. We calculated the mean time kestrels spent on foraging trips per day (both the time invested in commuting flights and in the foraging event) using complete days of tracking, using those dates and individuals in which we had continuous tracking data from sunrise to sunset. To estimate energy expenditure, we conservatively assumed that kestrels were resting when they were not on a foraging trip. Then, we estimated the individual daily energy expenditure from adopting a pure flapping or a pure soaring-gliding strategy in commuting flights during the breeding season. We considered 4.03 mLO_2_/min as the resting oxygen consumption, a figure that has been determined empirically for the lesser kestrel by open-circuit respirometry [46]. To estimate energy expenditure we used the standard conversion coefficient of 20.1 KJ/LO_2_ [47], resulting in a resting metabolic rate for lesser kestrels of 1.35 W, which we adopted instead of the basal metabolic rate calculated using Pennycuick’s software. Finally, we calculated the individual total daily energy requirement by adding the estimates of daily energy requirements for foraging and resting.

### Statistical Analysis

We used Generalized Additive Mixed Models (GAMMs) to estimate the potential influence of solar radiation and thermal and orographic uplift in the flight variables of the lesser kestrel foraging trips. We analyzed two groups of models considering the two sets of meteorological predictors used: either solar radiation (in Wh/m^2^) obtained at 30-minutes intervals, or thermal uplift (m/s) and orographic uplift (m/s) estimated at 6-h intervals (Table 1). Maximum foraging trip distance from the colony, duration, and its mean values per 6-h interval were logarithmically transformed.

**Table 1:** Summary of statistical analyses of lesser kestrel flight variables divided in two categories: Use of thermals and daily patterns.

Analysis	Level	Sampling frequency	Response variables	Meteorological predictors	Other predictors
Use of thermals	Commuting flight	1-second	Number of thermals/distance, Accumulated ascent/distance, Total ascent/thermal event, Mean climb speed/thermal event	Solar radiation	Individual (Random), Commuting flight type
Use of thermals	6-hour intervals	1-second	Number of thermals/distance, Accumulated ascent/distance, Total ascent/thermal event, Mean climb speed/thermal event	Thermal uplift	Date (Random)
Daily pattern of foraging flights	Commuting flight	All	Maximum flight altitude, Cross-country speed	Solar radiation	Individual (Random), Commuting flight type, GPS sampling frequency
Daily pattern of foraging flights	Foraging trip	All	Maximum distance from the colony, Duration	Solar radiation	Individual (Random), GPS sampling frequency, Colony, Colony*Solar Radiation
Daily pattern of foraging flights	6-hour intervals	All	Maximum flight altitude, Cross-country speed, Maximum distance from the colony, Duration	Thermal uplift, Orographic uplift	Date (Random), Colony, Colony*Thermal uplift

We analyzed these categories at different levels (commuting flights, foraging trips, or 6-hour intervals) registered at 1-second or at all (1-second to 10-minutes) sampling frequencies, depending on the category. We list the flight variables modeled at every level of analysis and the predictors tested in those models. Response variables used at the 6-hour interval level were mean values per interval.

In the first group of models, we fitted GAMMs to every variable indicative of intensity or efficiency in the use of thermals (i.e., number of thermal soaring events and accumulated ascent per horizontal distance, and total ascent and mean climb speed per thermal soaring event) to assess whether they were affected by solar radiation, which we were using as a proxy for thermal updraft intensity. We fitted GAMMs to maximum flight altitude in order to test our hypothesis of increasing flight altitude with increasing solar radiation based on the fact that thermal updraft increases in depth with solar radiation. We also fitted GAMMs to maximum distance from the colony to test the hypothesis that with higher values of solar radiation the kestrels have higher potential energy gain to fly larger distances at a lower cost. As soaring can have a negative influence on flight speed, we fitted GAMMs to study the effect of solar radiation on the cross-country speed of commuting flights and the duration of foraging trips. We used a Gaussian distribution of errors and the identity link function to fit models to all flight variables tested as a response variable excluding maximum flight altitude, cross-country speed, and total ascent and mean climb speed per thermal soaring event; for these variables we used a gamma distribution of errors and the logarithmic link function (which were more adequate after exploration of model residuals). We included solar radiation as a continuous predictor and individual identity as the random factor of all models. In models fitted to flight variables at commuting flight level (maximum flight altitude, cross-country speed and the 4 variables of efficiency of thermal use), we included the commuting flight type as a categorical predictor with 2 levels (outward and inward flight) so as to assess potential differences in the flight behavior of individuals when leaving or returning to the colony. In models fitted to flight variables at foraging trip level (maximum distance from the colony and trip duration) we included the breeding colony as a categorical predictor with 2 levels and also its interaction with solar radiation because the landscape mosaic of land uses in the surroundings of the two colonies was completely different (agricultural and urban) and had an impact on prey availability, and possibly strong effects on the kestrels’ foraging strategy. Furthermore, we included the GPS sampling frequency as a correction factor with 5 levels because it could affect calculation of variables obtained from flights tracked at different frequencies (maximum flight altitude, cross-country speed, maximum distance from the colony and duration).

For a direct estimation of the influence of thermal and orographic uplift on lesser kestrel flight variables and to differentiate the influence of each factor on flight behavior, we built a second group of GAMMs using mean values of flight variables at 6-h intervals as response variables to match the temporal resolution of uplift estimations available from meteorological models. These models provide a more direct insight into the relationship between lesser kestrel flight variables and thermal and orographic uplift compared to previous models that used solar radiation as a proxy. However they are constrained by the lower temporal resolution of climatic models which is an important limitation considering diurnal fluctuations in uplift. We used a Gaussian distribution of errors and the identity link function to fit models to all mean flight variables used as response variables except in those fitted to mean maximum flight altitude where we used a gamma distribution of errors and a logarithmic link. Residual analysis indicated that this was the best error distribution. In these models we included thermal uplift as a continuous predictor and date (year and day-of-year combined) as the random factor because of the lack of independence between observations on the same day that usually belong to the same individual. We also tested orographic uplift as a continuous predictor in the model to evaluate if kestrels flew not only by thermal soaring but also using slope soaring, more dependent on wind conditions. Since Env-DATA provided us with two orographic uplift estimates, we built two models for every response variable, each of which included one of the predictors and which were subsequently compared to each other. The orographic uplift estimate included in the best model of these two was also included in the final model of every response variable (see model selection later in this section). We performed a Bonferroni correction to adjust the significance level when testing the effect of orographic uplift on all kestrel flight variables. As with the first group of models, we also included the breeding colony and its interaction with thermal uplift as predictors.

We applied penalized smoothing splines to the solar radiation or thermal uplift estimate in all the GAMMs in order to take account of any nonlinear response to the predictor. The degrees of freedom of the smoothing function were automatically selected using restricted maximum likelihood (REML) [48]. We followed the Akaike Information Criterion (AIC) for model selection since it indicates that the best model is that with the lowest AIC value. The best GAMMs for cross-country speed of commuting flights, maximum distance from the colony, and duration of foraging trips when using solar radiation as a predictor were those including the smoothed term of the predictor. For the remaining response variables, the best GAMMs were those including the linear effect of solar radiation or thermal uplift. We therefore fitted these variables to Generalized Linear Mixed Models (GLMMs) with the same distribution of errors and the same link function used to fit the GAMMs, and including the same predictors and random factors. We fitted the GLMMs following a backward-stepwise procedure, by removing non-significant predictors until only significant ones remained. The significance of the predictors was tested using likelihood ratio tests comparing the model with and without the predictor.

Statistical analyses were performed using R-3.0.2 software [49] fitting GAMMs and GLMMs using “mgcv” [50] and “lme4” [51] packages, respectively.

## Results

### Use of thermals

**Fig 3:**
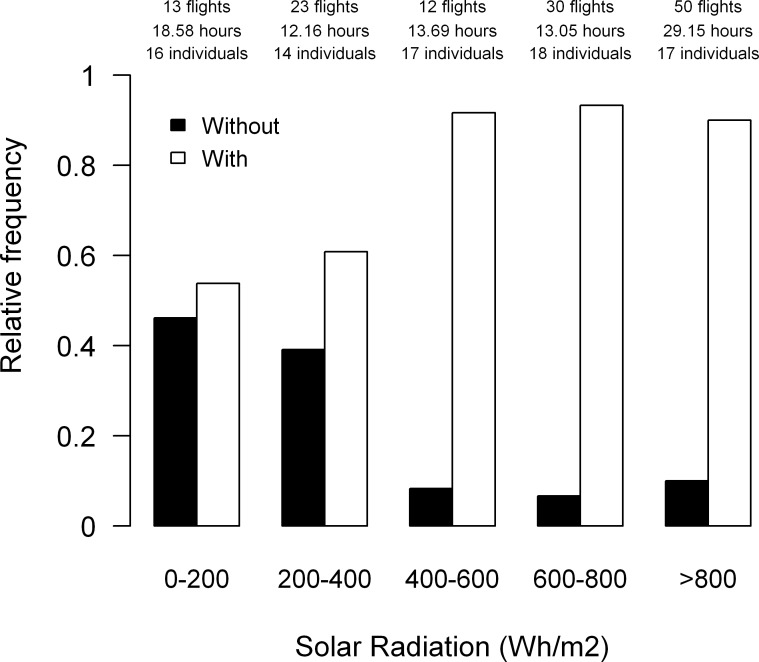
Relative frequency of commuting flights recorded at 1-second intervals with and without thermal soaring events in relation to solar radiation. Solar radiation is presented in categories of 200 Wh/m^2^. Numbers of commuting flights, tracking hours and tracked lesser kestrels per category are indicated above the bars.

We tracked 18 individual lesser kestrels (10 males and 8 females) with a GPS-datalogger programmed at 1 fix per second, and identified 303 thermal soaring events. Thermal soaring was present in 82.03% of commuting flights tracked at a 1-second frequency (n = 128, 64 outward and 64 inward flights). When comparing commuting flights with and without thermal soaring events we found that individuals flew at higher altitudes with lower cross-country speeds when using thermal soaring along commuting flights. Foraging trips were farther away from the colony and lasted longer when thermal soaring was used in comparison to trips where thermal soaring did not occur (Table 2). The frequency of commuting flights in which thermal soaring events were identified increased from approximately 55% at low solar radiation values to more than 90% at the highest values (Fig 3). Kestrel efficiency during thermal soaring in commuting flights is shown in Table 3. The models showed statistically significant positive effects of solar radiation on accumulated ascent per horizontal distance covered (β ± standard error = 0.05 ± 0.01, p<0.001), total ascent per thermal soaring event (1.34x10^-3^ ± 3.24x10^-4^, p<0.001) and mean climb speed per thermal soaring event (6.83x10^-4^ ± 1.45x10^-4^, p<0.001). The number of thermals per distance covered did not vary with solar radiation intensity (1.36x10^-5^ ± 1.34x10^-4^, p = 0.71). We did not find any effect that could be attributed to commuting flight type (inward vs. outward) on the response variables (p > 0.25).

**Table 2:** Parameters of lesser kestrel commuting flights with and without thermal soaring events tracked at 1 fix per second.

Flight variables	With	Without	Statistic	p-value
Maximum altitude (m)	193.56 ± 179.21	40.88 ± 36.89	Z = - 6.18	< 0.001
Duration (min)	10.80 ± 8.73	3.10 ± 2.32	Z = - 5.61	< 0.001
Maximum distance (km)	3.51 ± 3.01	1.32 ± 0.82	Z = - 4.76	< 0.001
Cross-country speed (km/h)	21.13 ± 9.43	26.53 ± 8.53	t = 2.70	0.01

We used the Mann-Whitney U test or Student’s t test to compare between commuting flights with and without thermal soaring events. Mean value ± standard deviation are shown. Sample size = 105 commuting flights thermal soaring events and 23 commuting flights without thermal soaring events.

**Table 3:** Parameters of lesser kestrel commuting flights with thermal soaring events tracked at 1 fix per second.

Flight variables	Mean ± SD	Min	Max	N
Number of thermals/distance (events/km)	0.64 ± 0.34	0.08	2.06	105
Accumulated ascent/distance (m/km)	59.44 ± 32.91	6.02	228.60	105
Total ascent/event (m)	123.07 ± 126.96	10	914	303
Mean climb speed/event (m/s)	1.37 ± 0.66	0.26	3.56	303

When considering 6-h intervals with flights recorded at 1 fix per second (n = 28), thermal uplift showed a statistically significant positive effect on mean values of all flight variables relating to thermal use efficiency (Table 4).

**Table 4:** Estimates (slope ± standard error) of the GLMMs fitted to 6-hour interval mean values of flight variables recorded in commuting flights with thermal soaring events tracked at 1 fix per second.

	Flight variables			
Predictors	Mean # Thermal events/distance	Mean Accumulated ascent/distance	Mean Total ascent/event	Mean Climb speed/event
Thermal Uplift	0.08 ± 0.03 *	15.22 ± 6.01 **	22.94 ± 10.71 *	0.15 ± 0.06 *

Mean values ± standard error are shown. Statistically significant variables are shown in bold

* p<0.05

** p<0.01

Sample size = 28.

### Daily patterns

**Fig 4:**
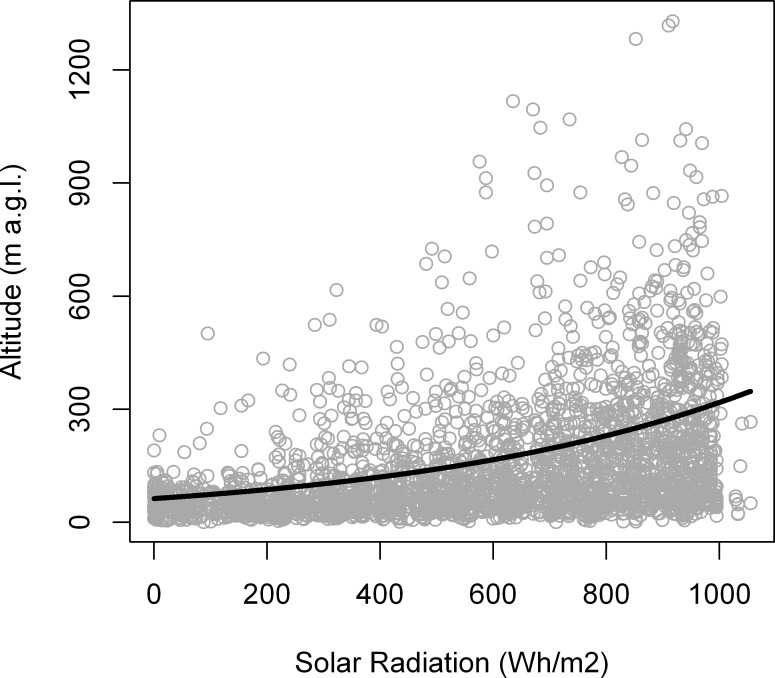
Effect of solar radiation on maximum flight altitude of lesser kestrels along commuting flights predicted by the GLMM. Circles represent the observed maximum altitude of commuting flights and the solid line represents the model prediction. Sample size = 2891 commuting flights.

**Fig 5:**
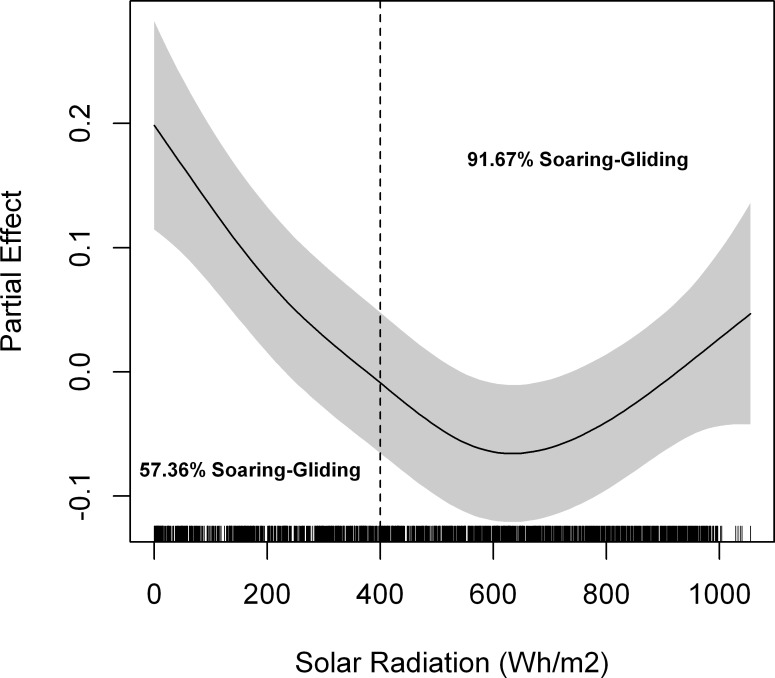
Partial effect of solar radiation in the model fitted to mean cross-country speed of lesser kestrel commuting flights. A penalized smoothing spline of 3.12 degrees of freedom was adjusted to solar radiation. Grey shading represents the standard error of the mean effect. Sample size = 2891 commuting flights.

The flight parameters of lesser kestrel commuting flights and foraging trips recorded at all sampling frequencies are summarized in Table 5. On average individual kestrels flew at a maximum altitude of 149.70 ± 164.42 m (mean ± standard deviation) with a cross-country speed of 21.13 ± 9.43 km/h along commuting flights. GLMMs showed a statistically significant positive influence of solar radiation on maximum flight altitude (0.002 ± 5.08x10^-5^, p < 0.001; Fig 4), as we had hypothesized. Neither commuting flight type (p = 0.15) nor GPS sampling frequency (p = 0.12) showed statistically significant effects on maximum flight altitude. The best GAMM fitted to cross-country speed included solar radiation and GPS sampling frequency (Table 6). Cross-country speed showed a negative curvilinear response to solar radiation, initially decreasing but then increasing as solar radiation values rose (Fig 5).

**Table 5:** Parameters of lesser kestrel commuting flights and foraging trips tracked at all sampling frequencies.

Level of Analysis	Variable	Mean ± SD	Min	Max
Commuting flights	Flights per individual	82.60 ± 87.16	2	354
Commuting flights	Cross-country speed (km/h)	17.06 ± 8.24	1.08	81.21
Commuting flights	Maximum altitude (m)	149.70 ± 164.42	0.40	1330
Foraging trips	Trips per individual	61.20 ± 63.62	2	237
Foraging trips	Maximum distance (km)	3.63 ± 3.37	0.34	32.23
Foraging trips	Duration (min)	69.43 ± 79.20	3.28	624.30

**Table 6:** AIC values of GAMMs fitted to kestrel flight variables calculated from commuting flights or foraging trips tracked at all sampling frequencies.

Predictors	Cross-country speed ΔAIC	Maximum distance ΔAIC	Duration ΔAIC
Smooth(Solar) + Type + Frequency	13.03	-	-
Smooth(Solar) + Type	66.81	-	-
Smooth(Solar) + Frequency	**Best Model**	-	-
Solar+ Type + Frequency	59.42	-	-
Type + Frequency	103.99	-	-
Smooth(Solar)*Colony + Frequency	-	2.25	**Best Model**
Smooth(Solar) + Colony + Frequency	-	79.87	3.95
Smooth(Solar)*Colony	-	**Best Model**	31.66
Smooth(Solar) + Frequency	-	76.12	1.98 **(Second Best Model)**
Solar+ Colony + Frequency	-	23.43	20.99
Colony + Frequency	-	151.27	11.34

The predictors are classed as follows: Solar radiation as “Solar”, Commuting flight type (outward vs inward) as “Type”, GPS sampling frequency as “Frequency”, and Breeding colony as “Colony”. ΔAIC was calculated between the best model and each proposed model. The best model fitted for each kestrel flight variable is indicated in bold.

**Fig 6:**
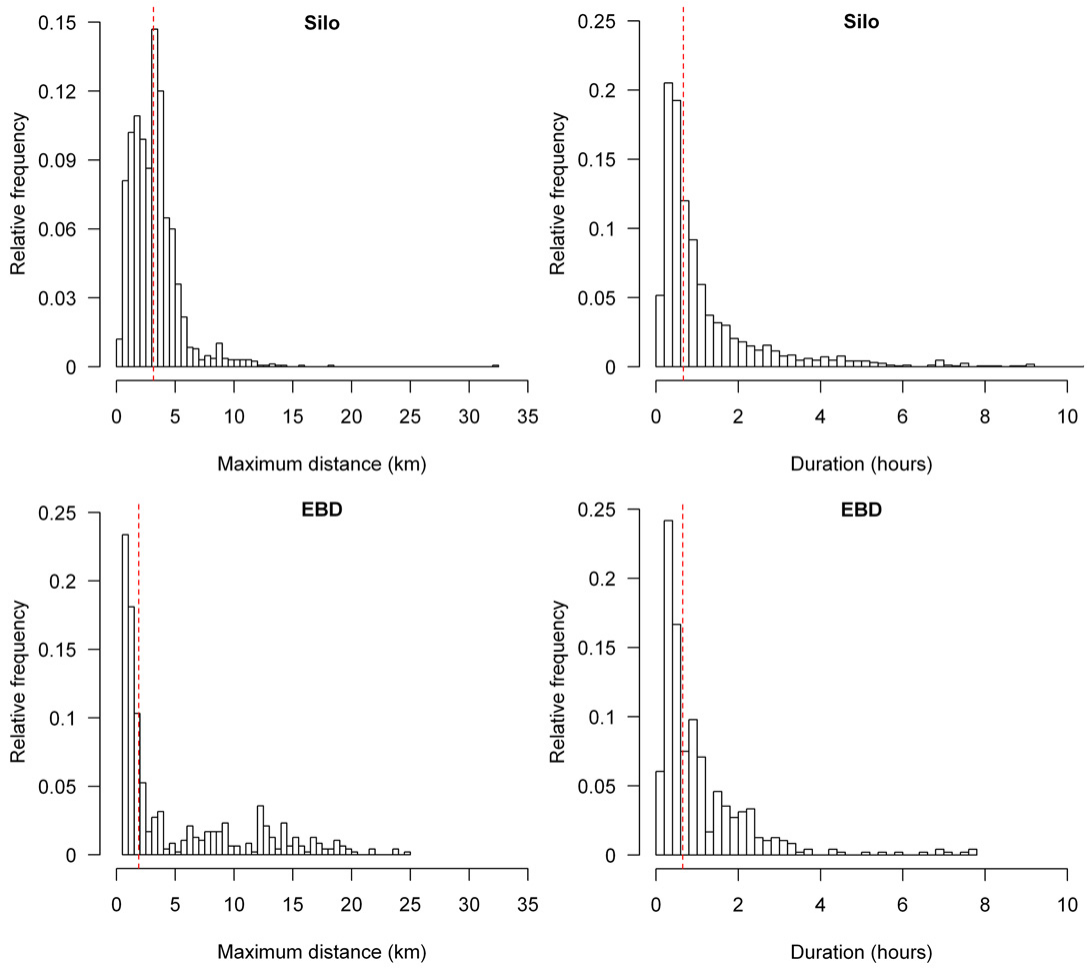
Frequency distributions of maximum distance (left panels) and duration (right panels) of lesser kestrel foraging trips from the Silo colony (upper panels) and the EBD colony (lower panels). The dashed lines represent the median value of flight variables. Sample size = 2142 foraging trips.

**Fig 7:**
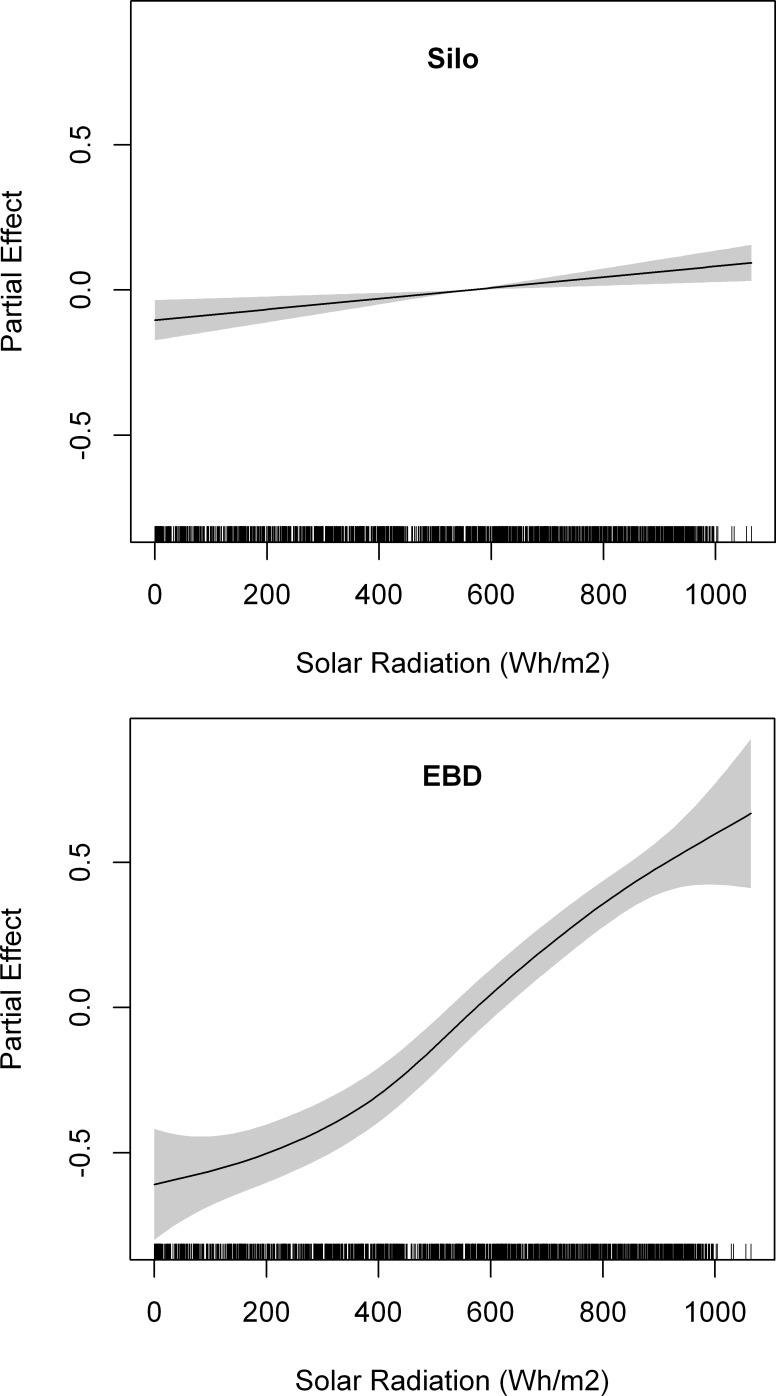
Partial effects of solar radiation on maximum distance from the colony of lesser kestrel foraging trips for individuals from the Silo colony (upper panel) and from the EBD colony (lower panel). Penalized smoothing splines of 1 and 2.72 degrees of freedom were adjusted to solar radiation for the Silo and the EBD colonies, respectively. Grey shading represents the standard error of the mean effect. Sample size = 2142 foraging trips.

**Fig 8:**
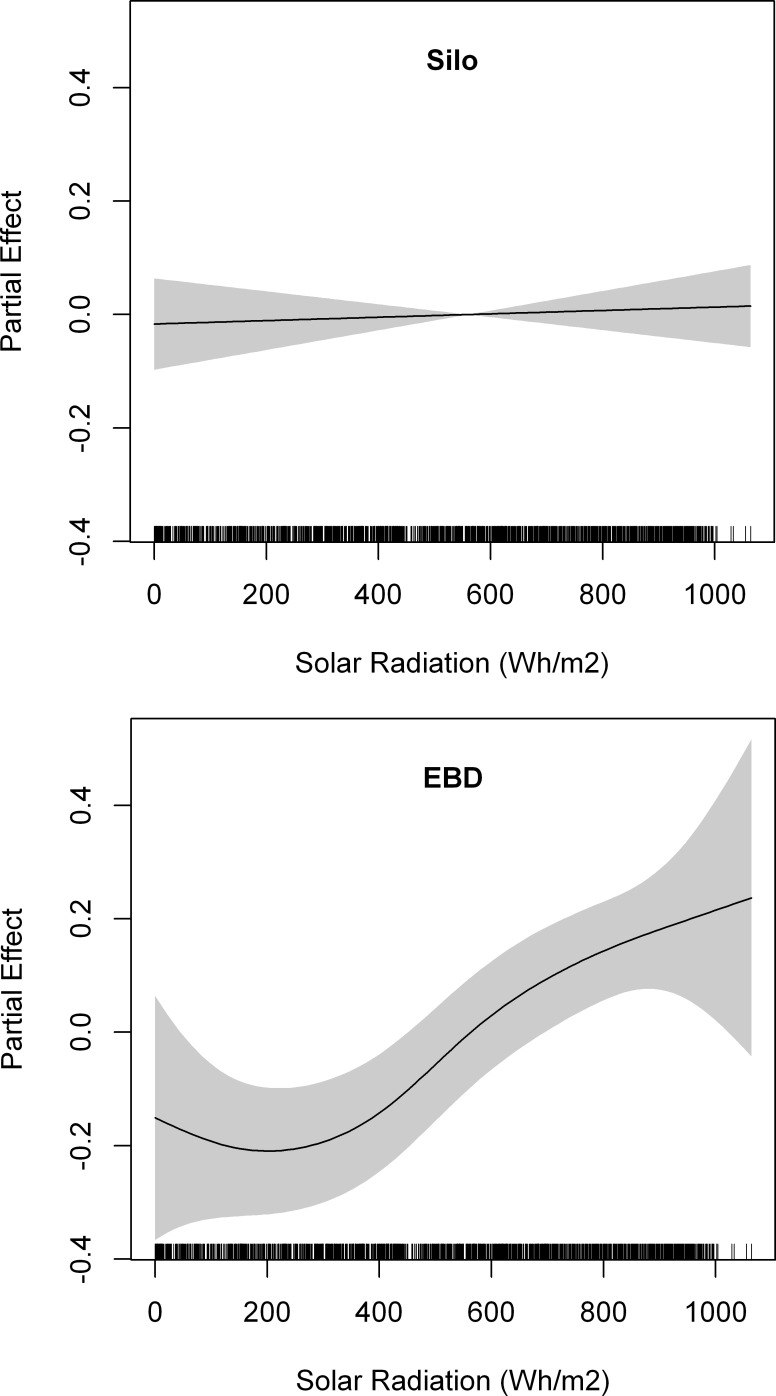
Partial effects of solar radiation on duration of lesser kestrel foraging trips for individuals from the Silo colony (upper panel) and from the EBD colony (lower panel). Penalized smoothing splines of 1 and 2.52 degrees of freedom were adjusted to solar radiation for the Silo and the EBD colonies, respectively. Grey shading represents the standard error of the mean effect. Sample size = 2142 foraging trips.

Lesser kestrels reached a mean maximum distance from the colony of 3.63 ± 3.37 km during foraging trips that lasted on average 69.43 ± 79.20 min. We did not find any statistical differences between breeding colonies on maximum distances covered (Mann-Whitney U test, z = - 1.60, p = 0.10), nor in duration (z = - 1.04, p = 0.30) (Fig 6). The best GAMM fitted to maximum foraging trip distance from the colony included the interaction between solar radiation and the breeding colony (Table 6). The interaction indicated that although maximum distance from the colony increased with solar radiation in both colonies, the response was steeper in the urban colony (EBD) with poorer foraging habitats than in the Silo colony (Fig 7), as we hypothesized. We obtained two different GAMMs fitted to foraging trip duration, as their AIC values differed by less than 2. Both models included GPS sampling frequency, but the best one included the interaction between solar radiation and the breeding colony whereas the second best model included only solar radiation (Table 6). One model showed that foraging trip duration increased almost linearly with solar radiation, while the other showed a steeper trend in the urban colony (EBD) than in the Silo colony, where the relationship remained almost constant (Fig 8).

When considering 6-h intervals with all flights regardless of the sampling frequency at which they were recorded (n = 533), thermal uplift showed a statistically significant positive effect on mean maximum flight altitude and maximum distance from the colony, but it did not show any effect on mean cross-country speed and trip duration. The interaction between colony and thermal uplift presented a statistically significant influence on mean maximum distance from the colony, but it did not influence mean duration. Orographic uplift presented a statistically significant negative effect on mean maximum flight altitude and a positive effect on mean duration, but no effect on mean cross-country speed and maximum distance from the colony (Table 7).

**Table 7:** Estimates (slope ± standard error) of the GLMMs fitted to 6-hour interval mean values of flight variables recorded in commuting flights or foraging trips tracked at all sampling frequencies.

	Flight variables			
Predictors	Mean Maximum altitude	Mean Cross-country speed	Mean Maximum distance	Mean Duration
Thermal Uplift	0.34 ± 0.03 ***	-0.02 ± 0.08	0.28 ± 0.04 ***	0.04 ± 0.02
Orographic Uplift	-1.29 ± 0.21 (ASTER)***	-0.48 ± 0.56 (ASTER)	0.68 ± 0.79 (SRTM)	2.47 ± 0.97 (SRTM) *
Colony (EBD)	-	-	-0.11 ± 0.08 ***	-0.002 ± 0.08
Colony*Thermal Uplift	-	-	-0.22 ± 0.05 ***	-0.10 ± 0.05

We indicate the orographic uplift model used to test its influence on each flight variable. Statistically significant variables are shown in bold

* p<0.25

*** p<0.001

Sample size = 533.

### Energy expenditure

Using Pennycuick’s Flight software we estimated 1.62 W of chemical power requirements for the lesser kestrel using a soaring-gliding flight strategy. Meanwhile, we estimated 5.02 W and 6.26 W of power required by the lesser kestrel using a flapping flight strategy at minimum power and maximum range speed, which were 31.36 and 54.72 km/h, respectively. Therefore, the power required for flapping flight ranged between 3.10 and 3.86 times the power required for soaring-gliding flight. Since the mean duration of the foraging trips was 69.43 min, the mean energy needed for kestrels to perform a foraging trip was 6.75 KJ when adopting a pure soaring-gliding strategy versus 20.91 KJ and 26.08 KJ for pure flapping at minimum power and maximum range speeds, respectively. The median time spent foraging per day was 7.48 hours; consequently the median time spent resting per day was 16.52 hours (n = 264 complete days). The median energy expenditure by the lesser kestrel during the breeding season would range from 124 KJ/day if using a pure soaring-gliding strategy in commuting flights to 249 KJ/day if commuting by flapping at maximum range speed. In Table 8 we provide our detailed estimates and calculation method.

**Table 8:** Estimates of daily energy expenditure (DEE) of a lesser kestrel for foraging, resting and total (foraging + resting) when using a pure soaring-gliding flight strategy or a flapping flight strategy at minimum power (Vmp) and maximum range (Vmr) speeds.

	Power Resting	DEE Resting	Power Foraging	DEE Foraging	Total DEE
Daytime		16.52 hours/day 14.26–18.11 9.29–24		7.48 hours/day 5.89–9.74 0.00–14.71	24 hours/day
Soaring-Gliding	1.35 W	80.29 KJ/day 69.30–88.01 45.15–116.64	1.62 W	43.62 KJ/day 34.35–56.80 0.00–85.79	123.91 KJ/day 122.36–126.10 116.64–130.94
Flapping at Vmp	1.35 W	80.29 KJ/day 69.30–88.01 45.15–116.64	5.02 W	135.18 KJ/day 106.44–176.02 0.00–265.84	215.47 KJ/day 194.45–245.32 116.64–310.99
Flapping at Vmr	1.35 W	80.29 KJ/day 69.30–88.01 45.15–116.64	6.26 W	168.57 KJ/day 132.74–219.50 0.00–331.50	248.86 KJ/day 220.75–288.80 116.64–376.65

We show median values of energy expenditure, the first and third quartiles, and the range, from top to bottom within each cell.

Sample size = 264 complete days.

## Discussion

Lesser kestrels, like other falcons, have traditionally been considered flapping raptors [17,26,31–35]. They are frequently observed to be hovering when foraging [52], they can fly at night when thermals are not available [26–28], they do not concentrate in big flocks over straits during migration as typical soaring raptors do [34], and they cross large water bodies where thermals are weak or absent [29,30]. However, our results show that lesser kestrels rely heavily on thermals and use them to soar in more than 80% of commuting flights between the colony and foraging areas during the breeding season. Unlike GPS tracking, direct observations are probably biased. While hovering kestrels are clearly visible in the field, a small kestrel gaining altitude on a thermal at more than 1000 m cannot be observed with the naked eye [35] (see S1 Video). Our research is a valuable example of new insights into bird flight strategies thanks to continuous tracking and the higher spatiotemporal resolution provided by recent bio-logging devices [53,54]. According to current state of flight theory it was not expected that small-sized birds, such as falcons, would rely heavily on thermal soaring because of the low energy benefits obtained given the cost in flight speed [16]. Nevertheless, our results indicate that lesser kestrels fly at relatively high cross-country speeds when soaring on strong thermals, a factor that to a certain degree mitigates the trade-off between energy and time when deciding to use thermal soaring. This is not the first study to describe thermal soaring in small birds [8,23,55], but our study indicates that thermal soaring is used by lesser kestrels in a similar way to that characterizing large soaring raptors [11]. It could be argued that the extra load of the GPS-datalogger (6 g) forces the kestrels to use thermals more often than normal. If extra load was the cause of frequent thermal soaring we would expect a difference between inward and outward commuting flights. In inward flights kestrels usually return to the colony with a prey which is approximately an average 1–20 g of extra load. The results obtained from testing the commuting flight type as predictor (Table 1) indicated no significant differences between inward and outward flights (Tables 6 and 7).

During foraging trips in which thermal soaring is used, lesser kestrels fly towards foraging areas located farther from the colony (2.5 times) than during those without, but at the greater cost of time (3.5 times). Moreover, kestrels fly at higher altitudes (4.5 times) with lower cross-country speed (20% slower) when they use thermal soaring along commuting flights than when they do not (Table 2). The use of thermals by kestrels increases with the availability and strength of thermal updrafts as observed by its increased use with solar radiation (Fig 3). Individual efficiency of thermal soaring (mean climb speed, total ascent per thermal and accumulated ascent per distance covered) increases with solar radiation (Table 3). Analyses, with a larger sample size, using all foraging trips throughout the breeding season also indicate that maximum flight altitude (Fig 4), maximum distance from the colony (Fig 7), and duration (Fig 8) increase with solar radiation. Cross-country speed in commuting flights initially decreases with solar radiation, but then increases again when the highest solar radiation values are reached (Fig 5). Our analyses show a consistent positive effect of solar radiation and thermal uplift on lesser kestrel flight variables that suggests that atmospheric kinetic energy is highly significant in kestrel foraging strategies. In contrast, we do not find any evidence of kestrels using slope soaring when commuting between the colony and foraging areas. This is not surprising due to the low relief of our study area and the absence of strong constant winds, but lesser kestrels could take advantage of slope soaring to fly in more abrupt areas, as other raptor species do[16,42]. As the availability, strength and depth of thermals are promoted by solar radiation, individual lesser kestrels use them more frequently and can improve their thermal soaring efficiency when solar radiation increases, as previously reported in large soaring raptors [23]. Our findings suggest a segregation of flight strategy of the lesser kestrel regarding solar radiation conditions. Kestrels seem to fly by flapping mostly at lower solar radiation intensities when thermals are weak or not available, but they prefer thermal soaring at higher values of solar radiation when thermals are stronger, in line with the flight strategy of European bee-eaters *Merops apiaster* [56].

Flight-cost models for lesser kestrels indicate that the soaring-gliding flight strategy is much cheaper (3–4 times) than continuous flapping. The difference is not negligible. This would explain why lesser kestrels mostly use thermal soaring when thermals are available. The increase in flight altitude with solar radiation (Fig 4) suggests an adjustment of kestrel flight strategy to thermal conditions in order to harvest the greatest possible amount of potential energy to reduce flight costs, as many studies have previously described in a variety of large soaring birds [22,57,58]. Considering that kestrels carry back one prey at a time and that optimal prey are 2–3 g grasshoppers [59], we estimate that long distance foraging flights for lesser kestrels would incur an energy deficit if flapping flights were used for commuting. A 2 g migratory locust *Locusta migratoria* (a typical prey species; see [57]) would provide 14.98 KJ [60]. In our study area the average foraging trip performed with flapping flight would cost kestrels 20.91 KJ at minimum power but 6.75 KJ with soaring-gliding. Consequently, kestrels would need to feed on three prey every two foraging trips in order to maintain a positive energy balance if individuals fly by flapping during the foraging trips, whereas they would need a single prey every two foraging trips when thermal soaring. Thus, thermal soaring becomes a cost-effective flight strategy for foraging kestrels, especially when individuals also have to feed their mate or offspring. However, using a soaring-gliding strategy increases flight duration because of the lower cross-country speed (Table 2). Accordingly, when using this strategy, kestrels are optimizing the energy balance at the cost of a lower chick provisioning rate at the colony. The cross-country speed at which lesser kestrels fly in commuting flights when not using thermals (26.53 km/h) is closer to minimum power speed (31.36 km/h), a figure that is far from maximum range speed (54.72 km/h) indicated by flight models. Thus, even when using the powered flapping flight strategy kestrels try to reduce costs by flying at the speed of minimum energy cost along commuting flights.

Thermal soaring is therefore an essential strategy for lesser kestrels to reduce flight cost when searching for food during the breeding season. Kestrels would develop a cognitive map of how prey are spatially distributed in the surroundings of the colony through direct experience or “public information” [61,62]. Individuals would overlay this cognitive map with thermal availability in order to decide where to go to forage and finally adopt the optimal flight strategy to be used after weighing up the trade-off between energy and time costs of the trip. This leads to the concept of energy landscape to describe the spatial distribution of movement costs regarding individual location [63]. The energy landscape of a central-place forager, such as the breeding lesser kestrel, is strongly affected by the distance required to commute between the colony, or central place, and the foraging area: the greater the distance, the higher the flight cost [64]. However, the energy landscape is not static and may change in space and time because of individual endogenous or exogenous factors [65]. Intraday variations in solar radiation mean that the energy available in the atmosphere in thermal updrafts is continuously changing in a predictable pattern. Therefore, there is a spatiotemporal energy landscape that kestrels can exploit on a daily basis. As solar radiation increases, prey that are farther from the colony have a lower energy cost. Thus, when thermal updrafts are low in the first hours after sunrise or before sunset, lesser kestrels adopt the costly flapping flight strategy to fly towards foraging areas close to the colony, resulting in short commuting flights. Meanwhile, as thermal updraft increases throughout the day, kestrels adopt the slower soaring-gliding flight strategy to fly towards foraging areas farther from the colony at reduced cost, but at the expense of a longer flight (Figs 3–5 and 7).

However, commuting to a foraging area far from the colony is only advantageous if prey are of higher quality in those areas, they are available in higher densities, or easier to catch. So, if kestrels increase foraging distance with solar radiation (Fig 7) or thermal uplift (Table 6) this is because foraging farther afield provides them with some advantage. Negative density-dependent effects, such as low prey availability or high intraspecific competition, are commonly experienced by colonial species in the surroundings of the colony [66,67] and might provide kestrels with enough motivation to fly towards foraging areas located far from the colony as soon as thermals form. As kestrels could easily reach these areas with flapping flight in the absence of thermals at a higher cross-country speed, this also supports the idea that there is an energy rather than a time constraint in increasing commuting flight distance. This is clearer when we compare the two kestrel colonies. The Silo colony surrounded by herbaceous crops, an optimal habitat for the lesser kestrel [68], shows a slight increase in foraging distance with solar radiation (Fig 7), while foraging trip duration remains almost constant (Fig 8). Lesser kestrels increase foraging distance with the help of thermals thereby reducing competition and prey depletion close to the colony only when they can maintain the same chick provisioning rate. The EBD colony, surrounded by poorer habitats is likely to suffer from greater competition or prey depletion. As soon as thermals are available lesser kestrels fly towards herbaceous crops far from the colony, causing a decrease in the chick provisioning rate. This explains the bimodal distribution in the maximum foraging trip distance from the colony (Fig 6), and the dramatic increase in that distance (Fig 7) and in foraging trip duration (Fig 8) with solar radiation. Therefore, thermal soaring is a crucial strategy for the lesser kestrel to prospect larger areas in the surroundings of the colony when searching for the unpredictable explosions of insects, especially when the colony is situated within a poor-quality habitat matrix.

Our estimates of daily energy expenditure for individual lesser kestrels during the breeding season (Table 8) overlap in range with those previously obtained for this species using doubly-labelled water (~ 300 KJ/day) by Tella [69]. The difference in average values could be due to the study period, since Tella [69] estimated mean daily energy expenditure of kestrels during the nestling period whereas our estimates relate to the whole breeding season. Indeed, our values were similar to the daily energy expenditure of common kestrels (*Falco tinnunculus)* for the entire breeding season (200–400 KJ/day) [70]. However, when we examine the estimates of daily energy expenditure in relation to the flight strategy in foraging, we observe that those adopting a pure soaring-gliding flight strategy are much lower. The reason for this may be because lesser kestrels are unlikely to complete foraging trips by adopting only a pure soaring-gliding flight strategy, as they usually hunt by hovering, while they can use a pure flapping strategy when thermals are not available. Consequently, daily estimations of energy expenditure when foraging with a pure soaring-gliding flight strategy would underestimate the real values. Accordingly, our estimations of the lesser kestrel’s daily energy expenditure when adopting one pure flight strategy or another establish the extreme values of the energy expenditure gradient, within which the real values would be located.

To conclude, lesser kestrels rely heavily on thermals for foraging flights during the breeding season. Our findings indicate that lesser kestrels show a temporal segregation of flight strategy that leads to a spatial segregation of foraging areas on a daily basis. Kestrels fly by flapping towards foraging areas close to the colony when thermals are absent, resulting in short foraging trips. But, as soon as thermals are available, kestrels use them to soar towards foraging areas far from the colony, presumably in order to avoid high competition, prey depletion or low-quality habitats in areas surrounding the colony, resulting in long foraging trips and consequently a reduced chick provisioning rate. This spatiotemporal segregation was more marked in the urban EBD colony, which is located in a poor-quality habitat. Our results indicate that during the breeding season lesser kestrels are more energy than time-constrained. The small size of the insect prey on which they forage and the limitation of providing a single prey at a time (kestrels transport a single prey to the colony in their beak or talons) mean that they can only forage far from the colony by harvesting energy from the environment, and at the expense of a reduced chick provisioning rate.

## Supporting Information

S1 Video Simulation of lesser kestrel flight during a real foraging trip tracked at 1-second frequency. Simulation has been produced using Doarama, an on-line 3D visualization engine that allows GPS tracks to be uploaded and uses aerial imagery from the Bing repository (https://www.doarama.com). In the upper left corner, Doarama offers some statistics of the foraging trip, from top to bottom: the flight speed, the accumulated distance out of the total distance traveled along the trip, flight altitude above sea level, climb rate between consecutive locations, and date-time information for the foraging trip. The bottom side of the frame shows the flight altitude profile throughout the trip.
(MP4)
